# Defining outcome measures of hospitalization for assessment in the Japanese forensic mental health scheme: a Delphi study

**DOI:** 10.1186/1752-4458-9-7

**Published:** 2015-01-28

**Authors:** Akihiro Shiina, Masaomi Iyo, Yoshito Igarashi

**Affiliations:** Department of Psychiatry, Chiba University Hospital, Chiba, Japan; Chiba University Center for Forensic Mental Health, Chiba, Japan; Department of Psychiatry, Chiba University Graduate School of Medicine, Chiba, Japan; Division of Law and Psychiatry, Chiba University Center for Forensic Mental Health, Chiba, Japan

**Keywords:** Forensic mental health, Delphi study, the Medical Treatment and Supervision Act, Hospitalization for Assessment, Outcome measure

## Abstract

**Background:**

A new legislation concerning forensic mental health was established by the Japanese Government in 2005, the “Act on Medical Care and Treatment for the Persons Who Had Caused Serious Cases under the Condition of Insanity,” or the Medical Treatment and Supervision (MTS) Act. Since it was passed, however, there has been broad controversy over Hospitalization for Assessment (HfA), the first stage of the MTS scheme.

**Methods:**

Following a comprehensive literature search to assemble a list of candidates, we conducted a Delphi study to establish standard outcome measures for HfA.

**Results:**

Five Delphi rounds were conducted by 19 panelists including medical practitioners and lawyers. A total of 139 items were accepted as outcome measures for HfA based on panel agreement.

**Conclusion:**

The Delphi study established a list of HfA outcome measures for the MTS act, which will contribute to the optimization of the new forensic mental health system in Japan.

**Electronic supplementary material:**

The online version of this article (doi:10.1186/1752-4458-9-7) contains supplementary material, which is available to authorized users.

## Background

Forensic mental health is a rapidly growing subspecialty in psychiatry which has received recent attention [1]. There is a global trend toward deinstitutionalizing patients with mental disorders, necessitating forensic mental health systems to develop accordingly [2]. As a result, many countries have established legislation for offenders with mental disorders that link together different disciplines [3].

For many years, however, Japan had no specific legislation for patients with mental disorders who had violated criminal laws [4]. Such offenders were subject to Official Involuntary Hospitalization (OIH) under the Mental Health and Welfare Law, a system completely detached from the criminal justice system [5]. The OIH scheme was harshly criticized by lawyers for lack of proper legal procedures protecting patient rights, while psychiatrists were concerned that many hospitals accepting OIH patients lacked adequate facilities and staffs [6].

To address these problems, the Japanese forensic mental health system was reformed with the enactment of a new legislation in 2005, the “Act on Medical Care and Treatment for the Persons Who Had Caused Serious Cases under the Condition of Insanity,” otherwise known as the Medical Treatment and Supervision (MTS) Act [7]. Under this new scheme, a person who committed serious crimes in a state of insanity or diminished responsibility would be dealt with under a specific judicial administrative framework. The public prosecutor presents an allegation. Then a District Court would make a judgment specifying the treatment of the offender. The judgment panel consists of one judge and one Mental Health Reviewer, a psychiatrist with Judgment Physician license (the national license for forensic mental health specialists). The panel can arrive at three possible verdicts: an order to hospitalize the offender for medical treatment; an order to care for the offender as an outpatient in the community; or an order not to treat the offender in the MTS scheme. The offender is then obligated to accept the special psychiatric care provided by the designated medical facilities and to submit to continuous supervision by a Rehabilitation Coordinator working in a probation office. Thus, for the first time, courts and officers of the Ministry of Justice are involved in the treatment of patients with mental disorders, signaling the beginning of a new era for forensic mental health services in Japan [8].

Temporary hospitalization of an offender for psychiatric examination and medical observation (usually for a few months) is called Hospitalization for Assessment (kantei-nyuin, HfA). A psychiatrist appointed by the court, named Examiner Psychiatrist, conducts a psychiatric evaluation of the offender and submits a report to the court [9]. A lawyer is appointed as an attendant to defend the offender during the judgment process [10].

In 2008, the Japanese government published a list of 239 mental hospitals accepting offenders for HfA [11], which was increased to 286 institutions in 2013 [12]. However, there have been no specific regulations regarding the conditions of the hospitals accepting HfA cases, and the availability of resources and the environment at these institutions vary markedly [13, 14]. Similar argument has occurred about the OIH setting, thus, some hospitals lack adequate resources for appropriate care of offenders with mental disorders [15].

Several studies have been performed in an attempt to optimize the HfA procedure [16–18]. We previously conducted a written questionnaire survey of leading Japanese forensic psychiatrists, to establish an expert consensus about how to deal with HfA cases [9]. We concluded that this consensus should be widely publicized among practitioners to ensure better management of offenders during HfA.

On the other hand, the goals of HfA are not clearly defined. The MTS act describes its aims as both assessment and medical observation of the offender, but there are no standards to evaluate the appropriateness of the medical care in each HfA. For example, there is a debate regarding which should be prioritized, neutral attitude against the offenders for accurate evaluation or development of the therapeutic alliance with them [19], so-called dual-role dilemma. Medical practitioners are struggling to determine how best to deal with offenders because of the lack of outcome indicators for HfA [20].

In light of this uncertainty, we conducted a study to define standardized HfA outcome measures to help practitioners determine the best treatment strategy for offenders. In addition, such outcome measures will be useful for evaluating the appropriateness of management in each HfA case subsequently, thereby contributing to the optimization of the system responsible for implementing the MTS act.

To define the outcome measures of HfA, we conducted a Delphi study, a method frequently used by multidisciplinary panels to establish a rational consensus on some issues not lending itself to precise analytical techniques [21–23]. Although there are many variations of the Delphi study protocol, it always involves a group of experts providing confidential ratings of agreement with a series of statements [24, 25]. Their answers are compiled, and returned to each rater for reference in subsequent rounds. Recurrent evaluations are used progressively to select the final list of the items which most agreed upon, which are thus moderate and trustworthy, compared to expert opinions merely gathered in single survey or conference [22].

## Materials and methods

### Study design

**Figure 1:**
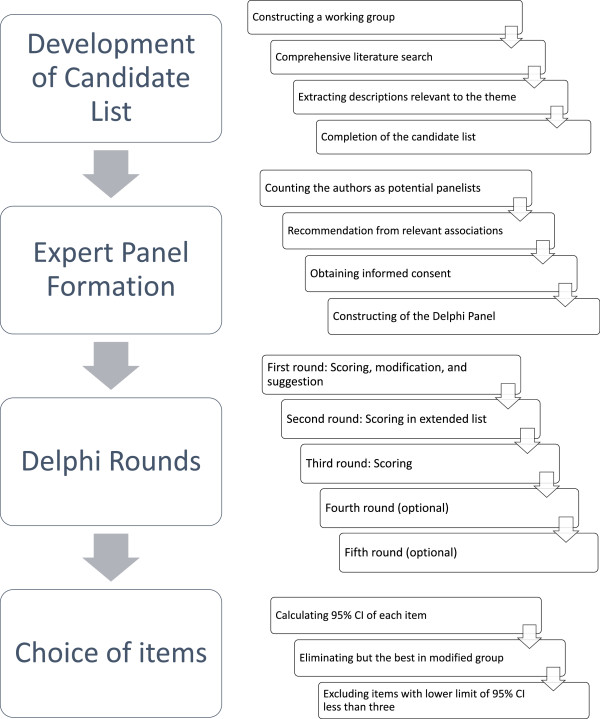
**Overview of the study protocol.**

This study consisted of several parts in establishing the outcome measurements. We proceeded the study according to the protocol developed in advance as follow: “Development of the candidate list”, “Expert panel formation”, “Delphi rounds”, and “Choice of items.” An overview of the study protocol is shown in Figure 1.

### Development of the candidate list

The purpose of this part was to create a list of candidate HfA outcome measures to be evaluated by Delphi panelists. For development of the list, we constructed a working group composed of some members belonging to Chiba University Center for Forensic Mental Health.

We searched the scientific literature using Igaku Chuoh Zasshi (Japan Medical Abstract Society, one of the biggest databases of Japanese medical journals; http://search.jamas.or.jp/) and the LEX database internet (one of the biggest databases of legal precedents in Japan; https://www.tkclex.ne.jp/) with the search term “kantei-nyuin.” We also searched PubMed using the search terms “MTS act” and “Japan.” All items retrieved were fully investigated.

According to this protocol, a total of 68 articles were retrieved from Igaku Chuoh Zasshi that included the words “kantei-nyuin,” of which 55 included some description regarding HfA outcome measures. Three articles were also retrieved from PubMed that contained the words “MTS act.” A total of 17 legal precedents including the word “kantei-nyuin” were found by searching the LEX database internet, of which 12 concerned the contents of HfA.

Additionally, we investigated the first 100 websites listed by Google in response to the search words “kantei-nyuin”, of which 10 referred to precedents regarding HfA, 15 were related to a hospital which accepted HfA cases, 6 were news items about HfA, 27 were opinions against the scheme of HfA, 17 were academic articles, 4 were official papers, and 5 were other than the above. Sixteen listed websites were not related to HfA.

We comprehensively investigated all these materials to extract any descriptions suggesting possible HfA outcome measures, such as the sentences related to “What is HfA?,” “Goal of HfA,” and “Outcome of HfA.” Each sentence was then rewritten into a short and concrete statement describing an outcome measure for HfA that could be answered with “yes” or “no” by an evaluator. Redundant content was either excluded or integrated into one item. Each item specified who was to evaluate that particular item (e.g., “You assessed the nature of mental disorders in the offender.” Evaluator: Examiner Psychiatrist).

As a result, 233 candidate HfA outcome measures were compiled into the first questionnaire (Q1) given to the expert panel. Of these items, 88 were to be evaluated by the doctor in charge, 6 by the Examiner Psychiatrist, 61 by a psychiatric nurse, 28 by the Rehabilitation Coordinator, 23 by the offender, 12 by a family member of the offender, 7 by the attendant lawyer, 5 by a representative of the designated hospital, and 3 by a post hoc survey committee.

### Expert panel formation

The panel members consisted of three groups: psychiatrists with a license of judgment physician, medical practitioners other than psychiatrists, and lawyers. We attempted to recruit a total of 15 to 35 panelists based on the standard Delphi method [21].

To make sure that the selecting process of panelists is objective and not arbitrary, we adopted a strict procedure described below. First, we counted the number of times each potential panelist was listed as an author on a paper identified in the literature search described above. Any candidates belonging to Chiba University Center for Forensic Mental Health were excluded because they were involved in the previous stage. As a result, 28 psychiatrists, 10 medical practitioners other than psychiatrists, and a lawyer, all referred to more than once in the articles gathered, were listed up to be potential panelists. Second, we attempted to contact each person according to the number of publications referring to him/her. In addition, we requested the Japanese Society of Forensic Mental health (JSFMH, http://jsfmh.org/) to recommend mental health practitioners as panelists. We also recruited lawyers who had multiple experiences of participating the MTS act as an attendant lawyer. We explained the content of our study to each potential panelist via telephone and asked for their participation. We sent an acceptance form and protocol paper to each person who committed to taking part in this study.

Finally, 10 psychiatrists with a judgment physician license and three medical practitioners other than psychiatrist (a psychiatric nurse, a pharmacist, and a Rehabilitation Coordinator) accepted the offer. In addition, 3 psychiatrists who were recommended as panelists by the JSFMH accepted the offer, and 3 lawyers with multiple experiences as attendants in the MTS act were also selected. Thus, final Delphi panel was composed of 13 psychiatrists, 3 medical practitioners, and 3 lawyers. All panelists provided written informed consent. The panelists were not informed of the names of the other panelists.

### Delphi rounds

The Delphi process was composed of three to five rounds.

For round 1, we sent Q1 to all panelists via email and asked them to score each candidate for outcome measure on a Likert Scale (1: not at all appropriate, 2: hardly appropriate, 3: undecided, 4: considerably appropriate, 5: extremely appropriate). We explained that good outcome measures should meet the criteria as follows; “If the answer to this item is yes, this case is evaluated as appropriately treated in the HfA”; “If the answer to this item is no, this case is evaluated as inappropriately treated in the HfA”; “The evaluator can answer this item with either yes or no”; “This item has clear and definite meanings”; and “This item is variable for each case.”

In addition, the panelists were encouraged to propose any other candidate outcome measures based on their own experience. Proposals for preferred evaluators (e.g., Examiner Psychiatrist, psychiatric nurse, attendant lawyer), and any modification of the contents were also welcome. Additionally, any other comments upon each candidate were accepted. Each new or modified candidate outcome measure was included in the second questionnaire (Q2).

After all the panelists replied, the means and standard deviations (SDs) for all original items were calculated. In addition to new or modified items, all comments provided by panelists, keeping anonymity, were included in Q2.

In the second round, the panelists completed Q2 as described, and the items means and SDs were recalculated. The procedure followed with Q2 was the same as that for Q1, excepting that panelists were not requested to make any additional proposal or modification.

In the third round, results of the second round were sent to all panelists and the items were re-evaluated as in the first and second rounds. We calculated the mean scores of all items; if the mean from round 3 divided by that from round 2 was between 0.95 and 1.05 (i.e., not markedly changed) in all items, a consensus was assumed. Otherwise, up to two additional rounds were conducted.

### Choice of items

Finally, we analyzed the results statistically. The 95% Confidence Interval (CI) for each item was calculated. In the modified group, only those items were retained of which the lower limit of 95% CI was the highest. We also excluded all items with a lower limit of 95% CI less than three. The remaining items were defined as HfA outcome measures.

### Ethical issues

This survey focused on the opinions of experts and thus did not gather personal information on any actual patients. All participants in this study gave written informed consent to take part in this study, following a full explanation of the protocol. This study was approved by the ethics committee of Chiba University Post Graduate School of Medicine (Aug 29, 2012, No. 365). We registered this survey on the Clinical Trials Registry (CTR) of the University Hospital Medical Information Network (UMIN, Tokyo, Japan), with the unique trial number UMIN000012554.

### Term of implementation

We began the survey in December 2013 and completed it in July 2014.

## Results

### The Delphi rounds

The Delphi rounds were conducted via email. The panelists responded to all of the questionnaires and completed all rounds of the Delphi process.

In the first round, all 233 items developed using the initial literature review were evaluated. The mean item score was 3.87 ± 0.54 and the mean of the SD was 1.18 ± 0.25.

In this round, we encouraged the panelists to propose new items to be included as candidate HfA outcome measures on subsequent Delphi rounds. In addition, we accepted any proposals to modify the descriptions of the original items. Each proposal was counted as a new item and added to Q2. The total number of items on Q2 was 413. Of these, 133 were to be evaluated by the doctor in charge, 25 by the Examiner Psychiatrist, 87 by a psychiatric nurse, 39 by the Rehabilitation Coordinator, 34 by the offender, 20 by a family member of the offender, 16 by the attendant lawyer, 9 by a representative of the designated hospital, 7 by a post hoc survey committee, 21 by multidisciplinary team (MDT), 21 by the care coordinator 21, and 1 by a psychiatric social worker.

Q2 was then sent to all panelists. All comments from the first round were also included so that other panelists could be informed about them.

In the second round, the mean item score was 3.62 ± 0.69, which was significantly lower than that on the first round [unpaired T test: degree of freedom (df) = 644, T = 4.82, P < 0.00001], and the mean of the SD was 1.06 ± 0.25. For all subsequent rounds, the panelists were requested to evaluate each item by referring to the previous score.

In the third round, the mean of the 413 items was 3.54 ± 0.80, and the mean of the SD was 0.87 ± 0.21. Of the 413 items, only 227 (55.0%) had a mean score between 95% and 105% of that on the second round, so an additional round was conducted according to the same protocol.

In the fourth round, the mean of the 413 items was 3.39 ± 0.86, which was significantly lower than that in the third round (unpaired T test, df = 824, T = 2.59, P < 0.01), and the mean of the SD was 0.87 ± 0.22. Only 220 (53.3%) had a mean item score between 95% and 105% of that in the third round, so an additional round was conducted according to the protocol.

In the fifth and final round, the mean of the 413 items was 3.32 ± 0.89, and the mean of the SD of them was 0.77 ± 0.17.

**Figure 2:**
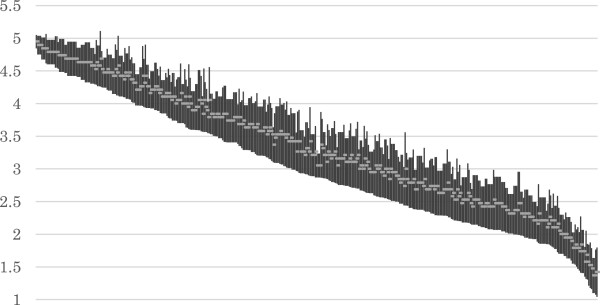
**The 95% CI of the each item.**

**Figure 3:**
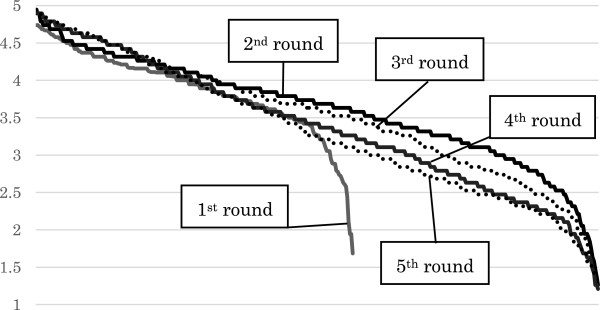
**Mean scores of the items in each round.**

The item means and SDs for each round are summarized in Table 1. The overall distribution of ratings for each item in the final round arranged by the lower limit of 95% CI is shown in Figure 2. The average scores of all the items in each round arranged by the mean score are shown in Figure 3.

**Table 1:** **Mean and standard deviation of the items in each round**

	1^st^round	2^nd^round	3^rd^round	4^th^round	5^th^round
Number of items	233	413	413	413	413
The average score of each item [mean ± standard deviation (SD)]	3.87 ± 0.54	3.62 ± 0.69	3.54 ± 0.80	3.39 ± 0.86	3.32 ± 0.89
The SD of each item (mean ± SD)	1.18 ± 0.25	1.06 ± 0.25	0.87 ± 0.21	0.87 ± 0.22	0.77 ± 0.17

### Choice of items

After completion of the Delphi rounds, we identified those items judged as highly appropriate HfA outcome measures from the list of candidates.

First, we eliminated all but those items with the highest lower limit of 95% CI among the group with modifications. For example, item No. 55 was originally “You assessed the risk factors in the offender (Evaluator: Doctor in charge).” In the Delphi rounds, three alternate evaluators were proposed by the panelists; MDT, care coordinator, and Examiner Psychiatrist. Then, all four candidates were scored in sequential rounds, resulted that each 95% CI was finally calculated as below: 3.95 - 4.68, 4.32 - 4.94, 4.08 - 4.77, and 4.75 - 5.04, respectively. Therefore, we determined Examiner Psychiatrist as the best evaluator, because its lower limit of 95% CI (4.75) was the highest among the four candidates. Other three candidates were eliminated. According to this process, 130 items were excluded.

Second, we excluded all items with a lower limit of 95% CI less than three, which eliminated 144 items from the list. As a result, 139 items were selected as HfA outcome measures (see Additional file 1: Table S1). Of these, 46 were to be evaluated by the doctor in charge, 17 by the Examiner Psychiatrist, 36 by a psychiatric nurse, 17 by the Rehabilitation Coordinator, 4 by the offender, 3 by a family member of the offender, 3 by an attendant lawyer, 6 by a representative of the designated hospital, 2 by an post hoc survey committee, and 5 by an MDT. There were no items retained whose evaluator was either the care coordinator or a psychiatric social worker, all proposed by panelists (see Table 2).

**Table 2:** **The number of items sorted by evaluator**

Evaluator	Q1	Q2	Adopted
Doctor in charge	88	133	46
Examiner Psychiatrist	6	25	17
Psychiatric nurse	61	87	36
Rehabilitation Coordinator	28	39	17
Offender	23	34	4
Family member	12	20	3
Attendant lawyer	7	16	3
Representative of the designated hospital	5	9	6
Host hoc survey committee	3	7	2
Multidisciplinary team	0	21	5
Care coordinator	0	21	0
Psychiatric social worker	0	1	0

Q1, questionnaire 1; Q2, questionnaire 2.

In the list, 114 items had unconditional descriptions (e.g., item No. 42 “You referred to the regulation of the MHW law to ensure adherence to it.” Evaluator: doctor in charge), whereas 25 were conditional ones (e.g., item No. 74a “[At a risk to harm self or others] immediate intervention was performed on the offender.” Evaluator: doctor in charge).

## Discussion

In this study, we attempted to establish an objective and comprehensive method for the evaluation of HfA outcomes, as no standard guidelines have been proposed by the Japanese government. We identified 139 items as appropriate HfA outcome measures using a Delphi study protocol.

In forensic mental health, outcome measures have proven to be difficult to establish due to the diverse nature of the subject and environmental factors. Recidivism is the most frequently adopted measured parameter in forensic mental health research [26]. However, many factors including recidivism were probably shown likely to be invalid as outcome measures [27]. Furthermore, recidivism may not be available as an outcome measure for HfA because the rates of recidivism of the crimes associated with the MTS act are usually too low to be statistically examined [8]. Therefore, we had to develop an original series of HfA outcome measures.

The Delphi method is widely used by expert panels to arrive at conclusions through Collective Intelligence [28]. It has been applied in many aspects of mental health, such as defining the feature of a particular syndrome [29, 30] and to evaluate community mental health care [31, 32]. An MDT is often appointed as a Delphi panel [33, 34]. Forensic mental health has also been utilizing the Delphi method [35], e.g., to establish guidelines for the treatment of psychosis in a local area [25, 36]. Attempts to identify quality indicators or professional roles are also performed using the Delphi method [37–39]. Therefore, we believe the Delphi method is appropriate for defining HfA outcome measures.

Repeated Delphi rounds made the list of candidates far more sophisticated. Mean scores of each item were gradually decreased over consecutive rounds (see Figure 3), but especially between round 1 and 2, suggesting that the panelists made a critical re-evaluation based on the comments made by other panelists in round 2. The individual item SDs were lower in later rounds, indicating evolving agreement on the appropriateness rating by the panelists. In summary, the judgments became more discriminating and uniform over successive Delphi rounds.

This study has some unique characteristics. We provided a lot of items which could be candidates of HfA outcome measures. Comprehensive literature search and exhaustive extract of relevant descriptions ensured that the list included the best evidence available regarding HfA optimization. Unlikely to typical Delphi methods, we did not set any criteria to dismiss items during the rounds. The reason is that we could not estimate the average scoring of each item in this study because there is no previous research to be referred in this topic. As a result, the panelists had to look through all of the items every time. Since the 139 items defined as outcome measures have particular 95% CI, we can compare the priorities of each item mathematically. For example, item No. 1001: “The relationship between the MDT and the offender was good.” (Evaluator: doctor in charge) has 3.04 - 3.91 of 95% CI, Instead, item No. 45a: “You identified the mental disorders existing at the time of the incident and the final decision.” (Evaluator: doctor in charge) has 4.42 - 4.95 of 95% CI. This means that the neutral observation should be prioritized than therapeutic alliance in this context, as an answer to the question described in the background section.

Limitations of this study include the dearth of source material (<100 articles) from which to develop candidate HfA outcome measures for the Delphi evaluation. This may have introduced bias in the final results. The list should be updated in the future in accordance with accumulating evidence on HfA.

In addition, we have not yet utilized the results for real cases under the MTS act. Some items have a room for much sophisticated in the clinical setting. It may be beneficial to compare the results obtained using this list with the opinions of professionals involved in specific cases by conducting face-to-face interviews.

## Electronic supplementary material

Additional file 1: Table S1: The outcome measures of Hospitalization for Assessment. (PDF 45 KB)

## Abbreviations

CI: Confidence interval
HfA: Hospitalization for assessment
JSFMH: Japanese society of forensic mental health
MDT: Multi disciplinary team
MTS: Medical treatment and supervision
OIH: Official involuntary hospitalization
SD: Standard deviations.
