# Supplementary material for: Defining outcome measures of hospitalization for assessment in the Japanese forensic mental health scheme: a Delphi study
Source: Int J Ment Health Syst. 2015 Jan 28;9:7. doi: 10.1186/1752-4458-9-7 (PMC4328080; doi:10.1186/1752-4458-9-7)
Supplement: Supplementary file 1 — Additional file 1: Table S1: The outcome measures of Hospitalization for Assessment. (PDF 45 KB) [file 13033_2014_220_MOESM1_ESM.pdf]

Supplementary Table: The outcome measures of Hospitalization for Assessment

Serial number	Evaluator	Item	Mean score	SD	95% CI
74a	doctor in charge	[At a risk to harm self or others] immediate intervention was performed on the offender.	4.84	0.37	4.67–5.01
75	doctor in charge	[At a risk to harm self or others] the hospital took an initiative in the intervention.	4.79	0.42	4.60–4.98
42	doctor in charge	You referred to the regulation of the MHW law to ensure adherence to it.	4.79	0.54	4.55–5.03
1009	doctor in charge	You carried out the necessary physical examinations.	4.79	0.54	4.55–5.03
34	doctor in charge	You clarified the nature of support for the offender to be reintegrated into the society.	4.74	0.45	4.53–4.94
40	doctor in charge	The limitation of psychiatric care was considered in the discussion about the judgment.	4.74	0.56	4.48–4.99
1006	doctor in charge	You explained your role at the beginning of the HfA.	4.74	0.56	4.48–4.99
1005	doctor in charge	You maintained good relationships with other physicians in case of physical complications.	4.68	0.48	4.47–4.90
45a	doctor in charge	You identified the mental disorders existing at the time of the incident and the final decision.	4.68	0.58	4.42–4.95
41	doctor in charge	You analyzed the relationship between the mental disorders and the incidence.	4.63	0.68	4.32–4.94
33	doctor in charge	You undertook measures to prevent the offender from committing suicide.	4.58	0.61	4.31–4.85
1008	doctor in charge	You carried out the necessary psychological examination.	4.58	0.69	4.27–4.89
39	doctor in charge	Enough information was gathered to make a diagnosis.	4.58	1.02	4.12–5.04
20a	doctor in charge	You undertook measures to prevent the offender from committing suicide.	4.53	0.84	4.15–4.90
16a	doctor in charge	[In the case of staying in the hospital where the HfA was done] the treatment plan was developed after the HfA.	4.47	0.51	4.24–4.70
3a	doctor in charge	You evaluated the alcohol or drug use of the offender.	4.47	0.77	4.13–4.82
21a	doctor in charge	You successfully prevented the offender from absconding.	4.47	0.84	4.10–4.85
31	doctor in charge	You summarized the treatment.	4.42	0.69	4.11–4.73
36a	doctor in charge	[If family members agreed with it] you gained the information necessary for treatment from family	4.42	0.69	4.11–4.73
38	doctor in charge	You assessed the responses of the offender with a psychiatric approach.	4.42	1.02	3.96–4.88
1007	doctor in charge	You assessed the offender on the basis of common evaluating factors.	4.32	0.67	4.01–4.62
15a	doctor in charge	The diagnosis of the offender was certified with good understanding of his or her mental state.	4.26	0.65	3.97–4.56
44	doctor in charge	You diagnosed the offender using operational criteria.	4.26	0.81	3.90–4.63
2	doctor in charge	You considered whether any other treatment than that mentioned by the MTS act is possible.	4.21	0.63	3.93–4.49
24	doctor in charge	You assessed and treated the offender at the beginning of the HfA.	4.21	0.71	3.89–4.53
7	doctor in charge	The examiner psychiatrist was a different person than the doctor in charge.	4.21	0.79	3.86–4.56
30	doctor in charge	As a principle, therapy was carried out with the consent of the offender.	4.16	0.50	3.93–4.38
22a	doctor in charge	You successfully performed some protection from problematic behaviors of the offender.	4.16	0.83	3.78–4.53
88	doctor in charge	[In the case that the institution where the offender stayed had to be changed] the process of transportation was carried out without troubles.	4.11	0.66	3.81–4.40
76	doctor in charge	[In the case that the offender rejected the therapy] you held an ethics committee meeting in the hospital to discuss the fairness of involuntary treatment.	4.11	0.81	3.74–4.47
17a	doctor in charge	You made a summary of the case before the end of the HfA.	4.05	0.78	3.70–4.40
18	doctor in charge	No negative incident occurred during the HfA.	4.05	1.13	3.54–4.56
5a	doctor in charge	You conducted a conference with the examiner psychiatrist in advance of the judgment.	4.00	0.67	3.70–4.30
28a	doctor in charge	You were aware of getting and administrating the information necessary for mental examination.	4.00	0.88	3.60–4.40
86	doctor in charge	[In the case of an incorrect allegation] you explained the incorrectness to the examiner psychiatrist.	3.89	0.74	3.56–4.23
6a	doctor in charge	You discussed with the examiner psychiatrist about each role, for better involvement in the case.	3.84	0.60	3.57–4.11
61	doctor in charge	You cooperated with other divisions in the hospital for security.	3.84	0.69	3.53–4.15
35	doctor in charge	You treated the offender with the knowledge that future treatment has not been established yet.	3.84	0.76	3.50–4.19
4a	doctor in charge	Personal information about the offender was reported to the hospital at the beginning of the HfA.	3.79	0.85	3.41–4.17
43a	doctor in charge	You explained the procedure of the HfA to the offender using special explanation papers.	3.79	0.98	3.35–4.23
14b	doctor in charge	The therapy was started on the first day of the HfA.	3.68	0.89	3.29–4.08
1	doctor in charge	You cooperated with a risk manager in the hospital.	3.63	0.76	3.29–3.97
11a	doctor in charge	A conference was held before the submission of the examination report.	3.58	0.84	3.20–3.96
13b	doctor in charge	The MDT was established before the beginning of the HfA.	3.58	0.96	3.15–4.01
1001	doctor in charge	The relationship between the MDT and the offender was good.	3.47	0.96	3.04–3.91
60	doctor in charge	You forecasted the prognosis of the offender.	3.37	0.60	3.10–3.64
54a	Examiner Psychiatrist	You assessed the nature of mental disorders in the offender.	4.95	0.23	4.84–5.05
2007	Examiner Psychiatrist	Adequate interviews were performed for medical examination.	4.89	0.32	4.75–5.04
55c	Examiner Psychiatrist	You assessed the risk factors in the offender.	4.89	0.32	4.75–5.04
69a	Examiner Psychiatrist	The responsiveness of the offender to the treatment was clarified.	4.89	0.32	4.75–5.04
2006	Examiner Psychiatrist	The examinations necessary to evaluate the mental state of the offender were safely performed in entirety.	4.84	0.37	4.67–5.01
56c	Examiner Psychiatrist	The risk factors in the offender were clarified.	4.84	0.37	4.67–5.01
58a	Examiner Psychiatrist	You assessed the responsiveness of the offender to the treatment.	4.84	0.50	4.62–5.07
2003	Examiner Psychiatrist	You had a conference with the MDT before submitting the examination report to assess common evaluating factors.	4.79	0.42	4.60–4.98
2002	Examiner Psychiatrist	Judge, prosecutor, mental health reviewer, and the attendant had a conference together before the judgment.	4.68	0.58	4.42–4.95
2005	Examiner Psychiatrist	There were adequate instruments to perform a physical examination.	4.68	0.58	4.42–4.95
46a	Examiner Psychiatrist	The relationship between the mental symptoms of the offender and the case itself was clarified.	4.68	0.95	4.26–5.11
2001	Examiner Psychiatrist	You discussed with the doctor in charge to share the information about the offender and to gain direction to perform the examination.	4.63	0.76	4.29–4.97
2008	Examiner Psychiatrist	You maintained contact with the rehabilitation coordinator through the HfA.	4.58	0.61	4.31–4.85
94	Examiner Psychiatrist	[In a case of offenders over 65 years old] you assessed the responsiveness to the treatment considering the the age of the offender.	4.47	0.51	4.24–4.70
53b	Examiner Psychiatrist	You assessed adequately the criminal responsibility of the offender.	4.47	0.90	4.07–4.88
2004	Examiner Psychiatrist	The staff conducted a conference to discuss the diagnosis and treatment of the offender before submitting the examination report.	4.21	0.63	3.93–4.49
57c	Examiner Psychiatrist	You forecasted how long would the motivation of the offender last.	4.05	0.71	3.74–4.37
101	psychiatric nurse	You observed and aided the offenders simultaneously.	4.79	0.42	4.60–4.98
100	psychiatric nurse	You observed in detail the behavior of the offender.	4.74	0.56	4.48–4.99
111	psychiatric nurse	You checked the security considering the risk of the offender acting out in the acute phase.	4.68	0.48	4.47–4.90
151	psychiatric nurse	[In case of problematic behavior by the offender] you reported the incident in detail to the concerned parties.	4.68	0.58	4.42–4.95
3001	psychiatric nurse	You regularly assessed the risk of the offender committing suicide, to share it with the team.	4.63	0.50	4.41–4.85
3002	psychiatric nurse	You regularly assessed the risk of the offender harming others, to share it with the team.	4.63	0.50	4.41–4.85
3003	psychiatric nurse	You assessed the risk involved in guiding the offender out of the ward, to share it with the team.	4.58	0.61	4.31–4.85
118	psychiatric nurse	You made sure that the staff understood the scheme of the MTS act well.	4.53	0.51	4.30–4.76
116a	psychiatric nurse	The MDT considered management of the daily life of the offender.	4.53	0.70	4.21–4.84
146	psychiatric nurse	The offender had been in a ward in which over one registered nurse per 13 inpatients was present.	4.47	0.51	4.24–4.70
115	psychiatric nurse	Symptoms of recurrence and the possibility of intervention in the case of exacerbation were well considered.	4.47	0.61	4.20–4.75
3004	psychiatric nurse	You explained well the meanings of restriction to the offender.	4.47	0.61	4.20–4.75
3005	psychiatric nurse	The MDT shared the information in case of seclusion.	4.47	0.61	4.20–4.75
113	psychiatric nurse	You considered the necessity and the degree of daily restriction.	4.42	0.69	4.11–4.73
126	psychiatric nurse	You intervened aggressively when the safety of the offender was at risk.	4.37	0.60	4.10–4.64
102	psychiatric nurse	You took care of the offender according to the records and reports gained from observation.	4.37	0.68	4.06–4.68
153	psychiatric nurse	[At a risk to harm self or others] the offender was transported or secluded to lower the risk.	4.32	0.67	4.01–4.62
143	psychiatric nurse	You determined several policies based on the consensus shared by the MDT.	4.26	0.65	3.97–4.56
136a	psychiatric nurse	You made a plan to care for the offender.	4.11	0.57	3.85–4.36
131a	psychiatric nurse	You assessed the medical care of the offender regularly.	4.11	0.74	3.77–4.44
132	psychiatric nurse	You assessed the offender through mutual communication.	4.11	0.94	3.68–4.53
135	psychiatric nurse	The HfA was held in a secure environment.	4.11	1.10	3.61–4.60
95a	psychiatric nurse	You ensured that the staff understood that it might have to deal with the offenses caused by the offender if necessary.	4.05	0.78	3.70–4.40
145	psychiatric nurse	You were prepared for carrying out the comprehensive violence protection program.	4.05	1.03	3.59–4.51
99	psychiatric nurse	You cooperated with psychiatric social workers.	4.00	0.58	3.74–4.26
103	psychiatric nurse	You clearly defined the goal of medical care at the beginning of HfA.	3.95	0.85	3.57–4.33
150	psychiatric nurse	[In the case of problematic behavior by the offender] you reported a solution which seemed to be beneficial based on the experiences from the offender's past.	3.89	0.66	3.60–4.19
148a	psychiatric nurse	Psychosociological treatment for a better life after discharge was planned.	3.89	0.66	3.60–4.19
128a	psychiatric nurse	You were involved in proper limited release of the offender from seclusion according to the directions of the doctor in charge.	3.79	0.54	3.55–4.03
149a	psychiatric nurse	[In the case in which a family member was the victim] you deliberately performed the intervention for the relationship between family members and psychological support for the family.	3.79	0.63	3.51–4.07
96	psychiatric nurse	You shared the policy and information regarding the HfA between medical and legal professionals.	3.79	0.71	3.47–4.11
98	psychiatric nurse	You cooperated with the rehabilitation coordinator.	3.79	0.71	3.47–4.11
129a	psychiatric nurse	The MDT conducted a conference with regard to treatment of the offender.	3.68	0.75	3.35–4.02
147	psychiatric nurse	More than 80% concerned with the HfA had training regarding the MTS act.	3.63	0.96	3.20–4.06
97	psychiatric nurse	You intervened with the family members for better understanding or resolving psychological distress.	3.53	0.77	3.18–3.87
114a	psychiatric nurse	Each nurse were conscious of his/her own role in the MDT in the treatment.	3.42	0.90	3.02–3.83
4003	Rehabilitation Coordinator	You gathered information about the community the offender belonged to, to clarify the risk factors.	4.79	0.42	4.60–4.98
159a	Rehabilitation Coordinator	[If necessary] application for public assurance was done during the HfA.	4.74	0.56	4.48–4.99
4001	Rehabilitation Coordinator	You gained the information about the life history of the offender from family members, to inform the examiner psychiatrist.	4.68	0.58	4.42–4.95
4004	Rehabilitation Coordinator	You explained well your role at the beginning of the HfA.	4.68	0.58	4.42–4.95
170a	Rehabilitation Coordinator	You shared the information about the problems associated with managing the offender in the past.	4.63	0.50	4.41–4.85
176	Rehabilitation Coordinator	The amount and quality of social resources for supporting the offender were clarified.	4.63	0.60	4.36–4.90
4002	Rehabilitation Coordinator	You conducted an interview with family members, to report it to the examiner psychiatrist.	4.63	0.60	4.36–4.90
163	Rehabilitation Coordinator	You made a comment from the point of view of reintegration of the offender into the society.	4.53	0.61	4.25–4.80
156	Rehabilitation Coordinator	The amount of the support of family and friends were clarified.	4.42	0.61	4.15–4.69
4005	Rehabilitation Coordinator	You explained the role of the family.	4.26	0.73	3.93–4.59
155a	Rehabilitation Coordinator	[In the case unlikely to be hospitalized] you prepared for the treatment to be administered after discharge from the HfA.	4.21	0.54	3.97–4.45
162a	Rehabilitation Coordinator	You cooperated with psychiatric social workers.	3.95	0.71	3.63–4.26
161	Rehabilitation Coordinator	You discussed with the attendant lawyer about the roles each other.	3.95	0.78	3.60–4.30
177	Rehabilitation Coordinator	The procedure of social support was established.	3.79	0.63	3.51–4.07
182	Rehabilitation Coordinator	[In the case of no treatment order] your case was handed over to a psychiatric social worker in a local institution to provide further support.	3.79	0.85	3.41–4.17
183	Rehabilitation Coordinator	[In the case of rejection of the appeal] your work was handed over to a psychiatric social worker in a local institution to provide further support.	3.58	0.90	3.17–3.98
160	Rehabilitation Coordinator	You supported the offender throughout the legal procedure.	3.53	1.12	3.02–4.03
206	offender	[In the case of a foreign offender] you had no trouble communicating by means of translators or other solutions.	4.05	1.08	3.57–4.54
5003	offender	You felt that you are free to post any letters anywhere without censorship.	3.74	0.99	3.29–4.18
5002	offender	You accept the necessity of medication with adequate explanation by the doctor in charge.	3.63	1.07	3.15–4.11
195	offender	You understood your rights and the legal procedure around you.	3.53	1.07	3.04–4.01
222a	family member of the offender	You could receive an explanation about the treatment policy by a staff member of MDT or the rehabilitation coordinator after being notified of the court decision.	3.79	0.85	3.41–4.17
218	family member of the offender	You got some explanation about the goal of HfA.	3.58	1.12	3.07–4.08
216b	family member of the offender	You were explained about the scheme of the MTS act in the early stage of HfA.	3.53	1.02	3.07–3.99
210	attendant lawyer	In the judgment, family members and the attendant lawyer made a comment about the treatment.	4.16	0.69	3.85–4.47
213a	attendant lawyer	You helped the offender understand the scheme of the MTS act and the MHW law.	4.00	0.94	3.58–4.42
6001	attendant lawyer	You had a meeting with the doctors to identify the problems around the offender.	3.84	0.90	3.44–4.25
230	representative of the designated hospital	[In the case of treatment order] the information about the offender was properly reported to the designated facility which would deal with the offender.	4.79	0.42	4.60–4.98
8003	representative of the designated hospital	[In the case of community treatment order] it was discussed whether it was possible for the offender to be hospitalized based on the MHW law.	4.05	1.03	3.59–4.51
228	representative of the designated hospital	[In the case of hospitalization order] you confirmed the decision was appropriate.	3.74	0.73	3.41–4.07
227	representative of the designated hospital	[In the case of community treatment order] you confirmed the decision was appropriate.	3.63	0.76	3.29–3.97
8001	representative of the designated hospital	[In the case of hospitalization order] you agreed with the opinion regarding the diagnosis of the offender.	3.63	0.90	3.23–4.03
229	representative of the designated hospital	[In the case of hospitalization order] the transportation was carried out without trouble.	3.63	0.96	3.20–4.06
29a	post hoc survey committee	It was confirmed that the discussion in the conference conducted before the end of HfA was considered into the final decision.	4.11	0.81	3.74–4.47
9001	post hoc survey committee	You discussed whether the treatment prescribed to the offender is justified.	3.47	0.84	3.10–3.85
37a	multidisciplinary team	You assessed the offender from a long-term point of view.	4.68	0.58	4.42–4.95
84a	multidisciplinary team	[In the case with high probability of no treatment order] you attempted to refer the offender to other therapists at a community level.	4.21	0.63	3.93–4.49
154a	multidisciplinary team	[In cases with a dual diagnosis] a conference was held to avoid any confusion around the diagnosis.	4.00	0.67	3.70–4.30
141a	multidisciplinary team	You made an effort to create and maintain a good relationship with the offender in the initial term of the HfA.	3.63	0.83	3.26–4.01
138a	multidisciplinary team	The MDT was involved in the treatment.	3.53	0.96	3.09–3.96
